# A Revisit of Plant Food Waste Along Food Supply Chains: Impacts and Perspectives

**DOI:** 10.3390/foods14081364

**Published:** 2025-04-15

**Authors:** Joana Gonçalves, Ofélia Anjos, Raquel P. F. Guiné

**Affiliations:** 1 CERNAS-IPV, Research Centre for Natural Resources, Environment and Society, Polytechnic University of Viseu, 3504-510 Viseu, Portugal; joanadgoncalves13@gmail.com; 2 Centre for the Research and Technology of Agroenvironmental and Biological Sciences (CITAB), Institute for Innovation, Capacity Building and Sustainability of Agri-Food Production (Inov4Agro), Universidade de Trás os Montes e Alto Douro (UTAD), Quinta de Prados, 5000-801 Vila Real, Portugal; 3 CERNAS-IPCB, Research Centre for Natural Resources, Environment and Society, Polytechnic University of Castelo Branco, 6001-909 Castelo Branco, Portugal; ofelia@ipcb.pt; 4 CBP-BI, Biotechnology Research Centre of Beira Interior, 6001-909 Castelo Branco, Portugal

**Keywords:** food waste, primary production, food processing, vegetable residues

## Abstract

More than one billion tons of the food produced in the world ends up being wasted every year, accounting for about one-third of the food produced globally. For this reason, the problem of food waste management has been the focus of the different actors intervening in the food supply chains, who recognize that food waste has not only environmental but also economic and social impacts. This review focuses on foods of plant origin wasted at different stages of their life, namely primary production, transformation/processing, transportation, sales, catering and the domestic level. It addresses the subject from multiple angles, considering the environmental, economic and social perspectives. The review was based on a search carried out within scientific databases, for example, ScienceDirect, Scopus and the Web of Science. The results highlighted that in the generation and management of food waste from plant origin, there is a clear difference between developed and developing countries, with these last showing higher losses in production, principally the transportation and storage of the foods. Contrarily, in developed countries, excess food produced and not consumed is the strongest contributor to food waste. Valorization of agricultural waste and industrial residues for application into animal feed or agricultural fertilizers, or through the recovery of valuable compounds for industrial purposes, are some of the ways to deal with food waste while generating additional economic value and reducing environmental impact. However, there is still a need to modify processes and behaviors to reduce food waste and improve the sustainability of supply chains. Therefore, it is crucial to conduct research to identify and report food waste so that stakeholders can contribute positively to solving this problem.

## 1. Introduction

Food waste is a critical global problem with significant environmental, economic and ethical impacts. The concept of food losses is, therefore, frequently related to post-harvest activities with a lack of system capacities or infrastructure [1]. On the other hand, the term “food waste” refers to “later stages of the food supply chain, such as retail and consumer households”. The FAO estimates that approximately one-third of the food produced worldwide is wasted (in general) or lost from production to consumption [2]. This estimate translates to around 1.3 billion tons of food wasted every year, which is equivalent to the production of 1.4 billion hectares of global fertile land, corresponding to 28% of the total agricultural area [3,4].

Food waste has harmful effects on many levels, such as economic, environmental, and social. It represents the utilization of resources within the food system, resulting in the emission of gases with greenhouse effects and a weakening of natural resources [5,6]. The carbon footprint of food waste is enormous and corresponds to the production of 3.3 billion tons of CO_2_ equivalent [7]. Additionally, food waste represents a cost of USD 750 billion to the global economy, with the FAO estimating that more than USD 936 billion worth of food is wasted each year [8,9]. Added to this figure are billions of dollars spent on transportation and appropriate disposal methods, and according to the FAO, a significant amount of food substance is wasted in almost all forms of food collected, generated and used [10,11]. In 2016, USD 49.5 billion worth of food was wasted, which corresponds to approximately 3% of the world’s gross domestic product [8].

The world population has been growing, and it is expected to increase from the present value of around 8 billion people to nearly 10 billion in 2050. As a consequence, the demand for food will rise due to population increases, as well as the expansion of some economies providing people with a higher financial availability to buy food. This will certainly put more pressure on the global food supply system [8].

Food waste currently represents a global environmental and economic problem, requiring urgent intervention to control/reduce the amount of waste [5]. Indeed, large amounts of food waste are accumulated daily as a result of industrialization and poor management, which is why the implementation of new, innovative standards is crucial [5,8].

By 2030, global food waste is expected to reach 3.40 billion tons at current rates [5]. Thus, one of the targets of the Sustainable Development Goals set out in the 2030 Agenda for Sustainable Development adopted in 2015 is to reduce food loss throughout the supply chain and reduce food waste at the consumption and retail stages by 50% by 2030 [2,4]. In Europe, a reduction in food waste is also foreseen in the European Circular Economy Action Plan and in the directive amending the European Waste Framework Directive [2].

Products of plant origin, like fruits or horticultural products, are responsible for a huge amount of residues and products that are not considered suitable for commercialization. Some of these are unsuitable due to a loss of objective quality, such as aging or degradation, but others are discarded for other reasons. In fact, the strict aesthetic and quality standards imposed by retailers contribute to waste, as edible but slightly imperfect-looking products are often discarded [12].

Considering the urgent problem of food waste and the negative consequences it involves, the objective of this review is to focus on plant-origin food waste along food supply chains, from primary production—including transformation/processing, transport and sale—to the final point of the chain, including at the consumer level.

## 2. Methodology

**Figure 1:**
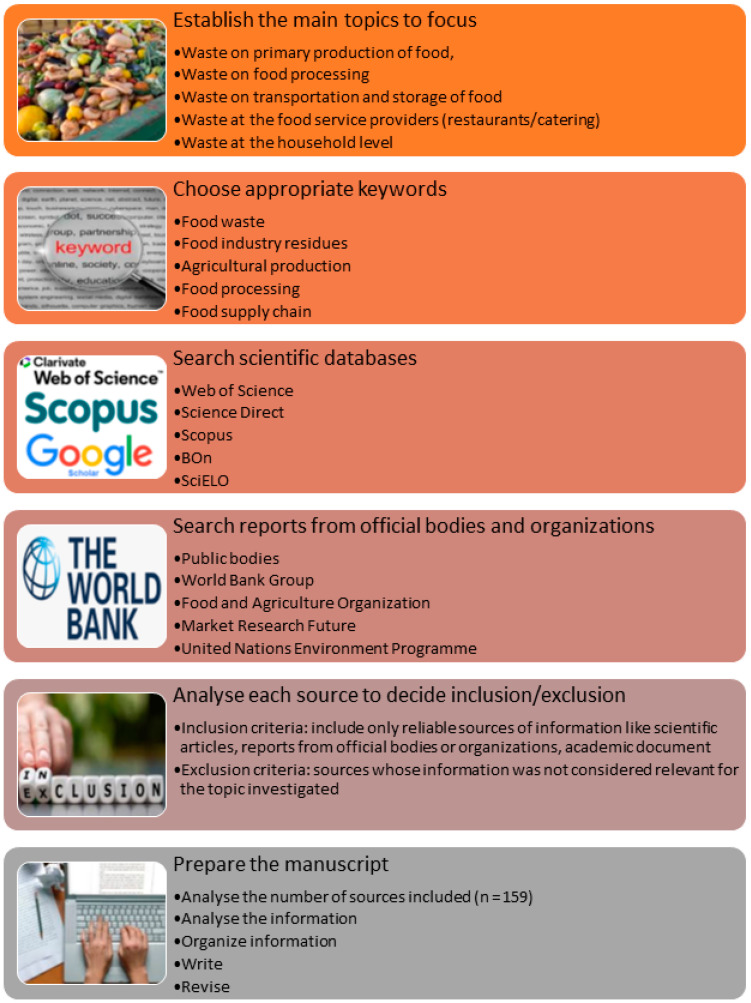
Schematic representation of the methodology utilized.

This is an integrative literature review, which started with a definition of the study object and the appropriate keywords to conduct the search on scientific databases (for scientific articles, books and chapters), as well as a search of reports from credible organizations that analyze the problem of food waste and its impacts. Figure 1 shows the phases applied to conduct this review.

## 3. Analysis of the Studies Included in the Review and Clustering of Co-Occurrences

**Figure 2:**
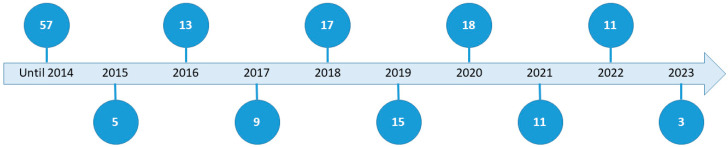
Distribution of the published works according to publication year.

This review is based on a total of 159 sources, of which 141 are scientific articles and 18 are different sources, such as books, chapters, reports or conference papers. Figure 2 shows the number of publications by year, showing a higher number of publications in the years 2018 and 2020, respectively, 17 and 18 publications.

**Figure 3:**
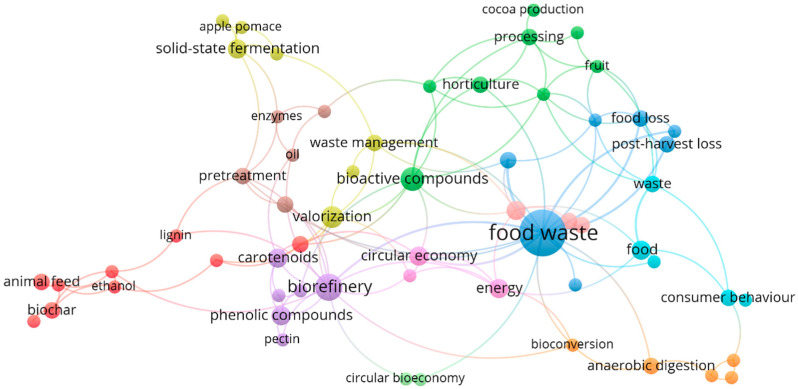
Network evidencing the co-occurrence links between the keywords that occurred at least twice.

The group of bibliographic references that corresponded to scientific articles (n = 159) was analyzed using the program VOSviewer, evidencing the type and frequency of keywords listed in each article. Figure 3 shows the link analysis based on the co-occurrence links between the 456 keywords. We selected the keywords that occurred at least twice (n = 61), and from these, one keyword was not related to any of the other keywords, so the final number considered to produce the diagram was 60. The size of the spheres in Figure 3 is proportional to the relative number of occurrences of each of the keywords, and the proximity of circles is related to the sources in which the keywords appear. The number of clusters formed by the keywords was 11, accounting for 130 links and a total link strength of 141. The most frequent keywords were ‘food waste’ (n = 23, link strength = 26), ‘biorefinery’ (n = 8, link strength = 15), ‘bioactive compounds’ (n = 6, link strength = 9) and ‘food loss’ (n = 3, link strength = 8).

**Figure 4:**
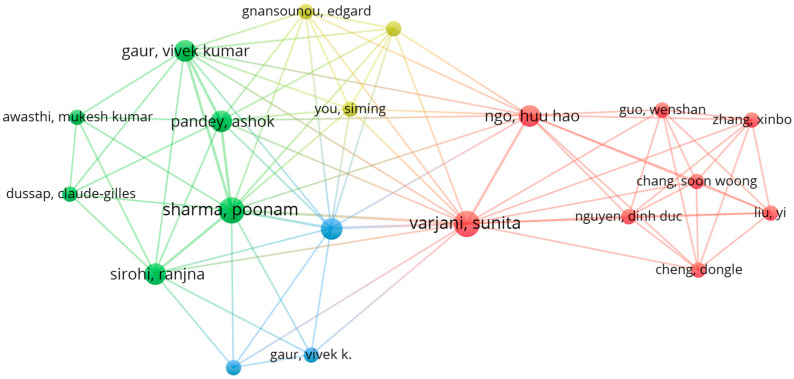
Network evidencing the co-occurrence links between the authors in the sources included in the review.

Figure 4 shows the links between the authors of the publications considered in the review, which were 652. From these, the maximum number of authors who appeared at least once and who were related to any other was 20. This group of authors formed 4 clusters, with 86 links and a total link strength of 94. The most frequent authors were VARJANI S. (n = 3, link strength = 20) and SHARMA P. (n = 3, link strength = 18).

## 4. Food Waste in Numbers

**Figure 5:**
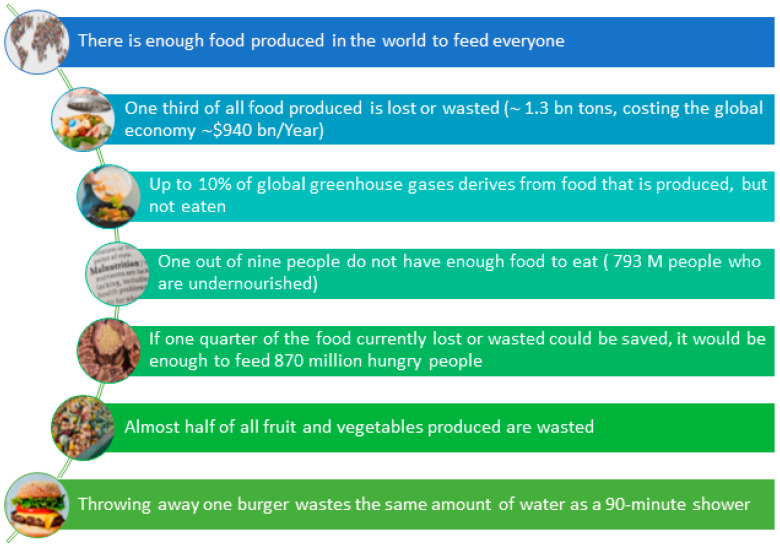
Food waste facts at a global level (Adapted from [13]).

While the growing world population poses a challenge for feeding everyone with quality and nutritive food, there is also a huge amount of food that is wasted while people are in desperate need of food. Figure 5 lists important facts about food waste at a global level, evidencing the high impact of food loss on a societal level as well as from an environmental point of view, evidencing that reducing food waste constitutes a highly effective way to address climate change [13].

According to the Food Waste Report of the United Nations Environment Programme [14], food waste represents failures at several levels: 1. Market: more than 1 trillion USD of food is thrown away every year). 2. Environmental: it is estimated that food waste generates 8–10% of global greenhouse gas emissions (including from both loss and waste), and it takes up the equivalent of ~30% of the world’s agricultural land. 3. Society: while food is being thrown away at a large scale, up to 783 M people are affected by hunger each year, and 150 M children under the age of five suffer from hindered growth and development due to a chronic lack of essential nutrients in their diets. It is, therefore, pivotal to implement effective measures to reduce food waste and recover food residues.

**Figure 6:**
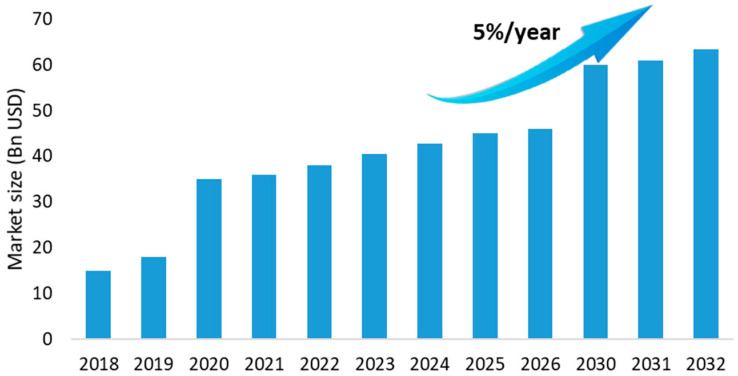
Food Waste Management Market (adapted from [15]).

It is expected that the Food Waste Management Market industry will grow from about 43 billion US dollars in 2024 to over 63 billion USD in 2032, representing a 50% increase over a decade (Figure 6) [15]. The increase is estimated to be 5.06% per year, and this is due to the expansion of the food service sector and the increase in food waste, which are estimated to be the main drivers contributing to the market growth and development.

The sectors contributing to the Food Waste Management Market include all the key actors in the food supply chain: Food Production Waste, Food Processing Waste, Distribution and Supply Chain Waste, Retail Waste, Consumption Waste and Household and Food Services. The waste generated at the household level was the highest, about 38% (14.5 billion USD) in 2022. It is envisaged that food waste generated in homes will increase as a consequence of not only the fast rise in the global population but also a modification of consumers’ behaviors. These include excessive purchasing, possibly driven by lack of time or fear of shortages due to unexpected external conditions, such as was verified during COVID-19; excessive quantities when preparing meals at home, possibly due to poor planning; inadequate skills for food management; and poor culinary skills [15].

**Figure 7:**
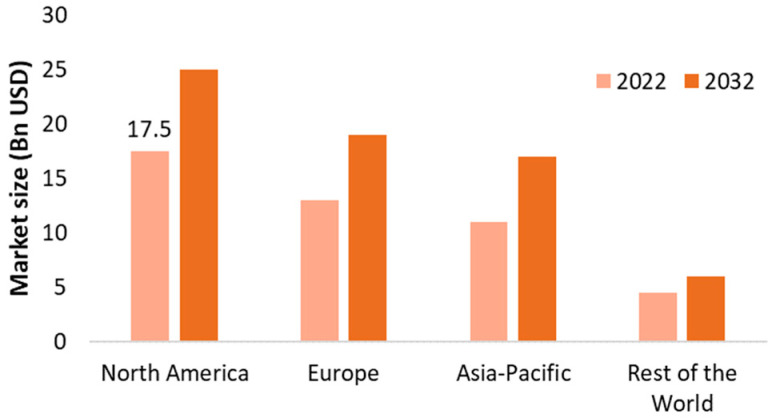
Food Waste Management Market by region (adapted from [15]).

Looking at the geographical distribution, it is expected that the North American market will dominate in the next decade. Reasons for this include an expansion of waste management assistance and activities at many levels, such as residential properties, institutional and business premises or manufacturing waste storage to minimize waste. Europe and Asia-Pacific regions will come next in market share, and the rest of the world represents a minor fraction (Figure 7) [15].

## 5. Primary Production

Waste associated with agriculture consists of leftovers generated during the production or cultivation of agricultural products or rural activities [16]. Rural activities also generate waste consisting of leftovers during harvesting and storage, namely the harvest of residues or spoiled or rotten crops [17]. This waste can have a major environmental impact, not only due to the waste generated but also due to the amount of resources used [18]. Subsequently, this environmental impact affects food security, interfering with food quantity, quality and accessibility and contributing to malnutrition in the population [18,19]. Additionally, food waste has a significant impact on climate change, biodiversity and health, contributing to a 60% increase in greenhouse gas emissions [19].

The generation of food waste is higher in developed countries, where 198.9 kg per capita is produced annually [19]. It is estimated that food waste is 20% in Europe and 32% in North Africa and West and Central Asia. The United States of America wastes 40% of the entire food production chain [19].

Separating the edible and inedible parts of food is sometimes tricky and very subjective, which can influence the classification of certain products as food waste [20]. Nevertheless, it is almost certain that, for most foods, particularly those of plant origin, waste will be produced at some stage in the supply chain, considering the perishable nature of such food products [21,22]. The application of effective waste reuse strategies can contribute to the reduction in the waste of these by-products [10]. Culture and eating habits influence this distinction between edible and inedible because while for some people, foods such as apple or potato peels or bread crusts are edible foods, for others, they are not [20]. Despite this, there is waste at all stages of the food supply chain, which includes production, processing, storage, transportation, sale and cooking, with waste depending on several factors [12,20]. In fact, there are some items that determine the degree of food waste, namely whether we are dealing with a developed or developing country, the climatic conditions and the occurrence of pests in crops, the efficiency of processing, transport and storage conditions, the visual appearance and demand for the product, and finally, the acceptance of the product by the consumer, as well as its accessibility to waste [23,24,25,26,27,28,29]. A large part of food waste comes from primary production sources, which include fruit and vegetable production or agriculture [5,20]. This is because fresh food is more perishable and, therefore, has a shorter shelf life [12]. At this stage, food waste is referred to as food waste from primary sources [5].

### 5.1. Fruits and Vegetables

Fruits and vegetables account for 40 to 50% of global losses, with 54% of this waste generated at this stage of the supply chain [30]. In fact, even before food is processed, a considerable amount of waste is produced, consisting essentially of stalks, stems, leaves, roots, straw, seeds, pods and peels, which raises government concerns [3,31]. In addition to these by-products, primary food waste from agriculture is often due to damage caused by bad weather, disease or damage caused during handling, inadequate post-harvest storage, size, aesthetic standards, failure to meet retail criteria or quality standards, surplus production or lack of attractive prices with consequent cessation of harvesting [5,20,32].

Among the various food categories, fruits and vegetables are the most wasted category in both developing and developed countries [20]. According to the FAO, in 2018, more than 1.8 billion tons of fruits and vegetables were produced worldwide, with China, India, Turkey and the United States of America being the main producing countries [19]. Regarding the production of tubers, China also stands out as the top producer (39%), followed by Nigeria with a production of 30%, India with 14%, Thailand with 9% and Congo with 8% [33]. Statistics from the following years were affected by the spread of the COVID-19 pandemic, resulting in an expected decrease [19]. Regarding the most produced foods in this sector, products such as tomatoes, apples, onions, peppers, mangoes, guavas, carrots, eggplants, cucumbers, oranges and cabbage stand out [34]. These types of foods are more susceptible to spoilage, even during production, due to their sensitivity, fragile surface membranes, poor temperature resistance and moisture content [20].

**Figure 8:**
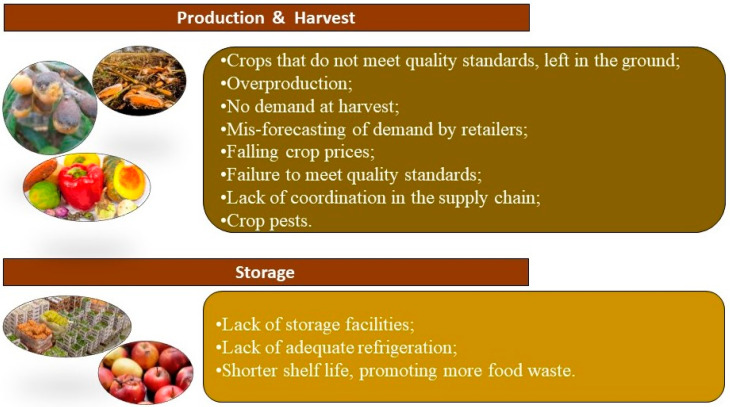
Causes of food loss in production and storage at the farm level.

Many factors account for the loss of agricultural crops in production and storage at the farm, as highlighted in Figure 8.

In developed countries, food waste from agricultural production can be very pronounced, reaching around 198.9 kg/year per capita [3,20]. Primary fruit and vegetable waste in Australia in 2020 and 2021 alone was around 2.5 million tons, with potatoes and tomatoes being the most wasted crops [5]. In the United Kingdom, potatoes were the most wasted crop in a list of 100 fruit and vegetable products, generating 10% (0.4 million tons) of total annual waste [35]. Another study revealed that in 2009, in Italy, 17.7 million tons of agricultural production (3.25% of total production) was left in the ground [36]. Also, in the United States, about 2.7 million tons of fruits and vegetables are not harvested or sold for aesthetic reasons every year [35]. On the other hand, in developing countries, the loss of fruits and vegetables varied between 15 and 50% in a post-harvest phase, as is the case in the Central African Republic [10,20]. In sub-Saharan Africa, about 10% of losses occur before harvest, while 8% occur during harvest [1]. Crops such as cassava and yam have a waste of 45% and 50%, respectively, on the African continent [37]. In the Philippines, papaya waste can vary between 30% and 60% of the total harvested [22]. In Rwanda, banana waste on farms was around 15% [1]. In short, Asia is the continent with the highest production of agricultural waste (47%), followed by the United States of America (29%), Europe (16%), Africa (6%) and Oceania (2%) [38].

Annually, 1.3 billion tons of waste are generated, corresponding to one-third of the world’s agricultural production [31]. Between 40% and 50% of the global loss corresponds to 520–650 million tons of tubers, fruits, vegetables and roots [39]. This waste is traditionally left in fields to rot or is incinerated, generating smoke and toxic gases, potentially carcinogenic, which, in addition to being harmful to health, contribute to the greenhouse effect [31]. However, residues from agricultural waste are usually nutritionally rich, as they have a high content of proteins, lipids and carbohydrates [40]. Thus, researchers have tried to develop a sustainable concept to value the residues generated by food waste [40]. In this way, waste can be used in the synthesis of value-added products or in the production of renewable energy [31]. In countries such as Australia, some strategies have already been used so that around 85% of food waste from primary production is left in the soil for its benefit, 11% is used in the manufacture of animal feed, and 4% is used in composting [5]. Another alternative for foods that do not meet sales requirements, such as size, presence of small stains, color or incorrect shapes, is to donate them to organizations that distribute food to people in need [32].

### 5.2. Grains and Cereals

Cereals also have a waste rate of 30%, and within these, grains are foods with a waste level of 15% of the world’s production [20,41]. Thus, grains constitute the second largest food group, after fruits and vegetables, yet their level of waste is significant [20]. Only in 2008, the United States of America produced USD 34.791 million in grain waste [21]. Rice is probably the most consumed grain, being considered the second most basic food in the world [20]. Even so, this cereal presents a waste level of 15% of the world’s production [20]. However, the information regarding grain waste does not specify which crops are most wasted; it only refers to grain waste in general, so it is not possible to understand which are the most wasted [20]. Despite this, post-harvest grain storage in developing countries has been studied, and it has been possible to verify that waste values are very discrepant between countries [20]. In southern and eastern Africa, grain waste levels amount to 13.5% of total production (USD 11 billion). In contrast, post-harvest and pre-processing cereal waste in sub-Saharan Africa costs around USD 4 billion [42]. Contrary to what was described, in Malawi, the level of grain waste is only 1% [20]. In other regions of the world, grain waste is also high, such as in Malaysia, where levels vary between 3% and 6%, in Bangladesh, where values are 12% to 13% or in China, where 19% of grain is wasted [20]. In other countries, the causes of waste are evident, as is the case in Pakistan, where 16% of total grain production (3.2 million tons) is wasted due to the lack of storage conditions and infrastructure, which leads to infestations caused by rodents [43]. India is one of the countries with the highest level of waste, accounting for around 30% of total production [44]. This level of waste costs an estimated USD 14 billion annually and is mainly due to a lack of storage facilities, poorly distributed warehouses, inadequate storage capacity and inefficient transportation and handling management [20]. In developed countries, there is a lack of studies on grain waste, but alternatively, this waste can be used to manufacture livestock feed [20].

### 5.3. Aquatic Plants

Aquatic plants have also been used for food, both human and animal, since 45 BC [20,45]. These plants are highly nutritious and sources of protein and polyunsaturated fatty acids [20,46,47]. Additionally, they have medicinal and antioxidant properties and can be used as dietary supplements [48,49,50]. Traditionally, algae and microalgae were consumed in Asia, particularly in Japan, China and Korea, but their consumption has expanded throughout the world [51,52,53]. Aquaculture production of aquatic plants is around 9 million tons; however, very little is known about the waste from these foods [20]. Nevertheless, some parts of these plants are used as raw materials for aquaculture and in the production of nutritional supplements for agricultural animals [20].

## 6. Food Processing and Transformation of Products of Plant Origin

Waste associated with food transformation and processing is related to increasing industrialization, which culminates in the production of inedible waste from food processing [54]. This processing at an industrial level leads to the production of such a quantity of waste that it is only surpassed by waste from domestic sewage [55]. Depending on the raw material, the industry processes various types of foods of animal origin, namely, cereals and legumes, fruits and vegetables, seeds and edible oils [19,31].

Industrial food processing inevitably generates primary and secondary waste. Primary waste consists mainly of organic waste composed of proteins, lipids or carbohydrates, for example, originating from different processing sectors such as fruits, vegetables and cereals [3]. Primary waste is obtained in the form of shells, shavings, seeds, bagasse or other vegetable residues [19,56]. Conversely, secondary waste comprises gases with a greenhouse effect, wastewater or packaging [18]. The agribusiness industry is a leader in the supply of food worldwide, but the release of residues such as plaster, dust, mist, acids, heavy metals or trichloroethylene, which are associated with several health problems, have been reported [31]. Solid primary waste can also cause problems at the ecosystem level later on, as it requires the use of high salt concentrations [57,58].

It has been reported that in Australia, over 90% of food waste comes from food processing, along with primary production and waste in businesses and households [5]. However, the vast majority of waste is associated with food and beverage processing, which between 2020 and 2021 alone produced 23.4 million tons of wasted food, with the sectors that contributed the most being the processing of fruits and vegetables, nuts, seafood, large-scale crops, wines and dairy products [5].

The amount of waste specific to each product may be unavoidable, and the reuse of waste may also be difficult at times due to biological stability, pathogenic potential, enzymatic activity or high water content [31]. Despite this, food processing allows us to increase longevity, as well as add value to the food products [31].

### 6.1. Processing of Foods of Plant Origin

The fruit, vegetable and cereal processing industries are those that generate the most food waste [59]. Around 46% of global fruit and vegetable losses occur in processing, along with the distribution and consumption phase [30]. Industrialization leads to the production of a considerable amount of leftovers after the processing of raw agricultural products, such as cereals and grains, fruits and vegetables [31]. Every year, peels, leaves, stems and seeds from the processing of juices (5.5 MMT—Million Metric Tons) and canned or frozen (6 MMT) vegetables and fruits are released into the environment [3]. Wine production produces around 9 MMT of waste, while grape juice produces 5 MMT per year [3,31]. Fruit and vegetable losses in the industry are around 60% of total production and between 3% and 50% of the processed product [19,60]. Regarding tubers, around 320 million tons are processed annually, resulting in waste of between 5% and 30% [19,33]. Despite this, the roots, tubers, fruits and vegetables industries are the fastest growing segments of agricultural production, which allows the marketing of products such as juices, jellies and dehydrated fruit, chocolate, beer, vegetable oils, flours and starches essential for the daily diet [19].

The processing of fruits and vegetables makes it possible to increase the shelf life of this raw material and generate other consumption options with greater value on the market, namely preservatives, juices, jams, pasta, concentrates and dehydrated products [61]. This production results in waste, such as peel, skin, seeds, stem, stone and pulp from industrial processes necessary for the processing and packaging of food [62]. Peels are generated when the pulp is separated in a peeling process, generating around 500 million tons of peel waste [63,64,65]. The seed is also highly wasted; it is generated during pulping, constituting between 15% and 40% of the total fresh weight of fruit [19,66]. Another waste that is generated in high amounts is bagasse, which consists of a mixture of peel, seeds, pulp and fibers generated during pulping and sieving [67].

Tomatoes are one of the fruits that contribute the most to waste during processing in Australia, with a loss of around 70% for the production of sauce or puree, generating tomato pulp (3% to 5% of the total mass) as waste [5]. However, several value-added products are produced from tomatoes, namely pastes, canned tomatoes, sauces, ketchup or purees [68]. Apples and citrus fruits also generate a large amount of waste. In Australia, when juice is produced, peels account for around 60% of the citrus fruit mass, and apple pomace accounts for around 30% of the mass [69,70]. Other studies also indicate that apple juice production generates around 11% of pomace as waste [71], and tangerine processing generates 16% of waste in the form of peels [31]. Orange processing generates 16 million tons of waste annually [65]. On the other hand, the production of papaya juice generates 15% waste, while its cutting produces 47% waste, of which 6.5% is seeds and 8.5% is skin. Additionally, 32% of the papaya obtained from cutting has imperfect cubes, which cannot be packaged and are, therefore, used in the juice [31]. In industrial pineapple processing, 9% of its mass is wasted in the core, 14% in the peel, 15% in unusable pulp and 15% in the upper part [31]. Industrially processed mango generates 11% peel, 18% unused pulp and 13.5% seeds, resulting in a waste of 42% of its mass [60]. In Australia, almond processing generates a significant amount of shells (85% of the total weight), making it the second largest producer in the world [5]. In Latin America, losses of fruits and vegetables during processing are around 30% and are mainly due to inefficient handling, storage and post-harvest logistics [30]. In the European Union, 89 million tons of food waste are generated annually, 39% of which comes from the manufacturing process [31]. In Mexico, the processing of vegetables such as beans, carrots, cabbage, lettuce and potatoes, cereals such as corn and fruits such as pears, tomatoes, lemons, oranges, bananas, pineapples, apples and papayas is responsible for producing 76 million tons of waste per year [72]. The most common waste generated is peels, seeds and pulp [73].

The tuber industry produces 820 million tons of products annually, which are preferably consumed in developing countries, as they have a low cost and high energy value [19]. Africa alone accounts for 15% of the world’s production (120 million tons), while Asia accounts for 13% and Latin America for 5% (135 million tons). Although tubers are consumed fresh, with the growth of industrialization, new products have emerged, including sweets, bakery products, frozen products, additives and snacks [19]. The flours produced have several applications, both in the industrial production of bread, instant soups and desserts and in the home-made preparation of cakes, tortillas and “arepas” [74]. Several by-products result from the industrial processing of tubers and roots. The husks represent between 2% and 15% of the mass and are generated in the peeling phase, representing an annual production of 11 million tons [74]. Bagasse is another by-product resulting from the phase in which the pulp is separated from the fiber and, in the case of cassava, represents around 10% of the latter [19]. Potatoes are among the tubers whose processing generates the most waste (around 25% of peels and 50% for chips) [75,76]. In addition to potatoes, tubers such as sweet potatoes, yams and cassava are among the most processed [77].

The cereal and pulse industries generate 429 million tons and 17 million tons of waste annually, respectively [31]. After fruit and vegetables, cereals and pulses are the foods that undergo the most processing, with China (cereals) and India (pulses) being the largest producers [19]. Wheat, corn, rice, sorghum, oats, cocoa, rye and barley account for a consumption of 2.5 billion tons, supplying most European, American and Asian countries [19]. As for legumes, beans, peas, chickpeas, pigeon peas and lentils are the ones that lead the production processes in the form of pre-cooked canned goods, additives, powders, condiments or instant soups [19]. The processing of cereals results in products such as cereal flakes, flours or edible oils [78].

Another cereal of great industrial interest is barley, which is mainly produced in the European Union, Canada, Ukraine, Australia and the United States of America [19]. Around 65% of its production is used in the manufacture of beer, with around 1.9 billion hectoliters produced annually, mainly in China [79]. This production results in the production of 475 million bags of bagasse [19].

Cocoa is also a widely cultivated crop, mainly in Africa, where 68% of world production occurs, and in Europe, Asia and America (15% of world production) [80]. Cocoa undergoes intensive industrial processing, and its powder is used to produce a variety of products, including butter, liqueur, sweets and chocolates [19]. The largest producer of chocolate is Europe (47% of the world market), followed by the United States of America [81].

The processing of cereals and legumes results in a large quantity of waste in the form of bran, germ, husk, endosperm, pulp residues, pellets, bagasse and broken grains [19,31]. Husks are generated in the initial stages of conditioning and in the peeling and milling phases, with the chocolate, agri-food, flour and vegetable oil industries producing the largest quantities of husks [79,82,83]. On the other hand, malt husk is mainly produced during the pressing and filtering of barley wort in beer production [19].

Residue cake is a by-product of the vegetable oil production process and was given this name because, after pressing, it produces a type of crushed cake, coarse flours and pellets [84,85]. China and the United States of America are the countries that generate the most waste during food processing, with the food industry responsible for 70% of global waste [19].

Waste generated by fruits, vegetables, cereals and legumes is biodegradable and rich in nutritional macromolecules, namely proteins, lipids, carbohydrates, fibers, minerals, vitamins, waxes, resins, oil, pigments and water [31]. Additionally, some bioactive compounds are also present in these generated residues, namely phenolic compounds, carotenoids, terpenes, β-glucans, glucosinolates and saponins, which have some biological properties, namely antioxidant, anti-inflammatory, cardioprotective, antidiabetic, antiobesity, immunostimulant, antimicrobial, anticancer and prebiotic [5,73]. Thus, this waste has great potential, but it is often disposed of in landfills or burned, which contributes to damaging health, animal life and the environment [19,38]. Landfills occupy an increasing amount of land and contaminate water and soil, so it is crucial to study the chemical composition of this type of waste and assess its potential for reusing raw materials in the production of new products, contributing to the circular economy [19].

**Figure 9:**
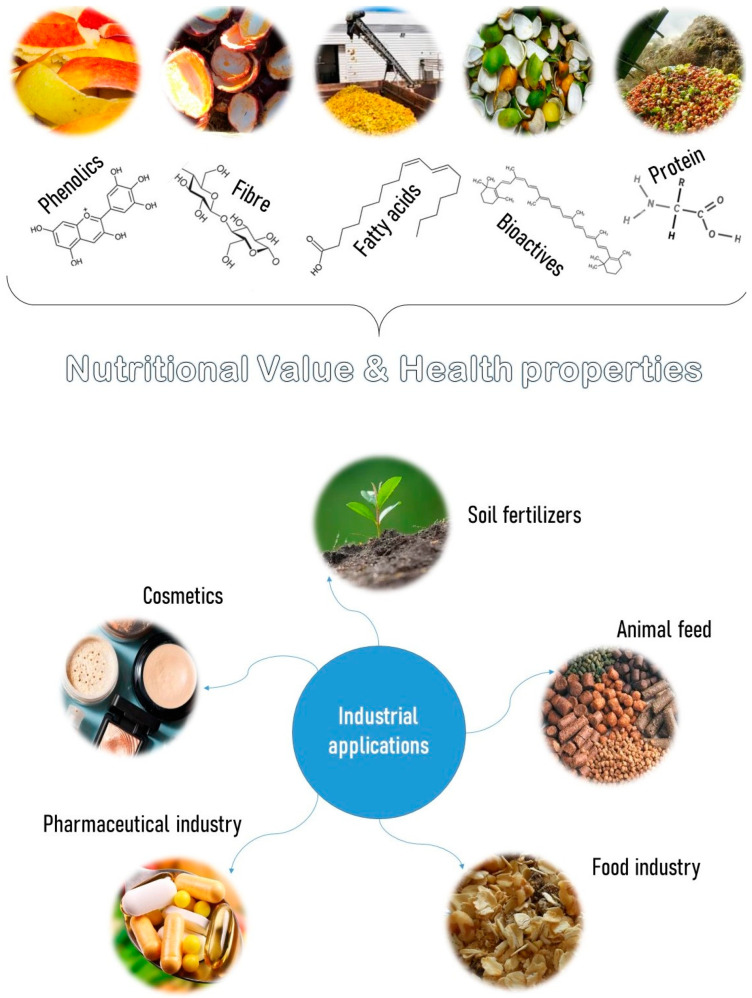
Reutilization of residues from the processing of fruits and vegetables.

Fruit peels are a source of phenolic compounds, peptides, terpenes, cellulose and hemicellulose [73]. These residues have antioxidant, therapeutic and nutritional properties and can, therefore, be used in the production of organic fertilizers, cosmetics and pharmaceuticals, as well as in the preparation of animal feed [62]. The fruit’s seeds also contain around 25% lipids and 35% proteins. Tomatoes generate a lot of seeds as waste, which are rich in linoleic acid, a carotenoid responsible for the fruit’s red color [19]. Mango seeds are an excellent source of phospholipids, phenolic compounds and fatty acids and can be used to extract edible oil [83]. On the other hand, fruit pulp contains between 40% and 60% fiber and other bioactive components. Grape pomace is a good source of catechins, anthocyanins, flavonoid glycosides, phenolic acids and stilbenes [86]. Meanwhile, pineapple seeds are a residue that can be used as a food source, being an excellent source of fiber [19]. Bromelain, an enzyme with anti-inflammatory properties, is also present in the seeds and can be used in the food industry, namely as a meat tenderizer, oyster sauce production, fish processing or as a substitute for sulfones used to prevent browning of fruit juices, white wine and beer [87]. This enzyme can also be used in the treatment of cancer, cellulite, edema or dyspnea, which is why it has applications in the pharmaceutical industry [88,89]. The peels of tubers are also used as animal feed, as they contain protein [19]. It is also possible to observe lignocellulosic characteristics in this by-product, which may vary depending on the product and the region where it was produced [19]. Cassava bagasse is rich in starch and water and has a high fiber content (22% cellulose, 35% hemicellulose and lignin), providing lignocellulosic characteristics [90]. The richness of industrial residues and byproducts generated by the processing of fruits and vegetables makes these a valuable source of components with nutritional value and health-promoting properties, which can be used in a variety of other products and serve as raw materials or ingredients for various industrial applications (Figure 9).

Table 1 presents some compounds that can be recovered from the food processing industry, which may have a potential for reutilization after recovery.

Cereal and legume husks also have a high content of cellulose and hemicellulose, with the highest content being found in corn, cocoa and beans [19]. Malt bagasse contains between 60% and 70% fiber and also proteins, carbohydrates and lignin [106]. Due to its composition, this by-product has the potential to be used in pastry, confectionery, animal feed or biorefinery [107,108]. Cereal and legume residue cakes are widely used in the production of animal feed due to their protein content [109]. Additionally, waste cake can also be used in the production of cereals, tofu, flour and sausages, which is why it is a waste widely imported by Mexico, the United Kingdom, the United States of America and Argentina [110]. Thus, waste from the processing of fruits, vegetables, cereals and pulses can be widely reused for the production of food, medicines or cosmetics [111].

Biorefinery and animal feed production have also emerged as alternatives to the disposal of these by-products [19]. These leftovers are a good way to obtain low-cost raw materials to produce higher-value products. Thus, ashes from rice husks, sugarcane bagasse and bamboo leaves have been used in the development of building materials, while rice husks, rice straw, peanut shells and coconut shells are used as partial substitutes for sand in the production of cement blocks [3].

### 6.2. Problems Generated by the Incorrect Disposal of Industrial Food Waste

The different industries in the food sector generate a considerable amount of waste [31]. As it has been possible to verify so far, the waste from food processing contains large amounts of biodegradable organic substances, namely proteins or nitrogenous matter, fat and carbohydrate content, carbon sources, total nitrogen and total phosphates [112]. The disposal of this waste entails significant economic costs and also has a significant environmental impact, contributing to acidification, eutrophication and global warming [113,114]. The most common method of disposing of this waste is in landfills, leading to the emission of hazardous gases, namely greenhouse gases [31]. In addition to the atmospheric implications, these gases can react with water to form nitric and sulfuric acid or ammonium nitrate, which evaporate and reach the soil later as acid rain [115]. The high amounts of salts generated by some sectors of the food industry interfere with soil permeability, reducing the value of irrigation and, consequently, affecting crop growth [31]. Soil quality can also be affected by wastewater from, for example, olive oil mills [31].

Waste from food processing has a high level of homogeneity, which means it can be reused to generate value-added products, particularly in biorefineries or in the production of chemical products [116,117]. Traditionally, these residues have been used in the production of fertilizers and animal feed [118]. Another important use attributed to these residues is their application as sources of renewable energy [39,119].

Some pesticides and herbicides present in agricultural waste are water-soluble and can cause contamination of water or other foods, leading to the development of Parkinson’s disease, birth defects, cancer, Alzheimer’s disease and reproductive disorders [120].

## 7. Transportation and Retail

Food transportation is an important step in the food supply chain. It is estimated that around 10% of fruits and vegetables are lost during transportation, as it is not always carried out in the most appropriate way, especially in less developed countries [1]. In sub-Saharan Africa, the main causes of fruit and vegetable losses are inadequate handling, storage and transportation [1]. Studies show that many of the losses are due to poor road conditions, which end up causing physical damage to the food. Poor storage facilities also lead to food losses, particularly tomatoes [121]. The installation of refrigeration systems, both in storage and during transport, is suggested as a measure to prevent losses, as it can slow down the proliferation of sprouts and the rotting of fruits and vegetables [1]. In India, between 18% and 40% of fruits and vegetables wasted annually are due to lack of refrigeration during transportation and storage, costing USD 71.481 million [20]. Apart from fruits and vegetables, poor storage and transportation are major reasons for grain waste in developing countries [122]. The overloading of transport vehicles also contributes greatly to food waste at this stage since mechanical pressure is increased, leading to the degradation of sensitive foods [1]. Another important focus aimed at reducing food losses during transport is the choice of resistant and suitable packaging [1]. In Ghana, losses of around 32% have been reported in cabbages transported in large bags [123]. During transport, this type of packaging does not protect the food and is easily damaged [1]. In Rwanda, bananas are often transported without packaging, leading to losses of 35% during this stage [123]. Also, in Ethiopia, papaya losses were mainly due to fruit rot, physical damage due to compaction, inadequate transportation and poor storage conditions, with losses of around 22% during wholesale, 12% during transportation and 9% during storage [124]. In Nigeria, 23.3% of tomatoes were lost during wholesale, with 23% of this being due to mechanical damage [125].

The use of cold storage is, in fact, the main method of avoiding losses of highly perishable foods and is the preferred method in many parts of the world [1]. However, in less developed countries, the use of cold storage is not always a solution since most require the use of electricity [126]. On the other hand, there are studies that report the use of refrigeration without electricity, namely hydroelectric cooling, evaporative cooling and forced air cooling, which are more suited to the reality of developing countries [127]. As already mentioned, the use of appropriate packaging is also crucial since cold storage only preserves food but does not prevent mechanical damage [1]. Furthermore, road networks are not suitable for the transport of some foods, and using appropriate packaging for each product can minimize the consequences of poor infrastructure [1]. Therefore, improved packaging is the best solution since it is easier and cheaper to implement [1]. When implementing packaging, it is important that the packaging has an appealing appearance, has little environmental impact and that the materials are inert when in contact with the food [1]. Studies carried out in some African countries and India have shown that the use of lined plastic boxes allows for a greater reduction in food damage compared to unlined boxes or wooden boxes and woven baskets [127]. The same was observed when replacing raffia baskets (41% losses) with plastic baskets, with a 5% reduction in damage [128]. In sub-Saharan Africa, choosing the right packaging reduces fruit and vegetable waste by 30% to 50% compared to traditional packaging [129]. In addition, packaging allows food to retain its water and nutrient content [1]. The same was verified with the use of Controlled Atmosphere Packaging, where it was found that the shelf life of belladonna improved, maintaining levels of protein, vitamins, chlorophyll, lycopene, lutein and *β*-carotene [1]. However, the production of highly efficient packaging is not within everyone’s reach, so the production of ecological, biologically based packaging using local materials appears to be an advantageous alternative for consumers, farmers and the planet [1].

Other inappropriate practices, namely product reception and hygiene and handling failures, have also been responsible for food loss [30]. Food products often present failures in hygiene conditions, leading to contamination risks, whether during display or handling, leading to losses or even causing illness [30]. Failures in the hygiene of transport vehicles also contribute to accelerated maturation and senescence of food, which can lead to food loss [30]. In short, hygiene, packaging and compliance with the maximum capacity of the packaging significantly reduce food losses without being highly costly [1].

Food loss during the sales process is also a concern. Lack of planning of quantities purchased for sale and excessive handling, associated with the packaging failures previously discussed, are identified as the main causes of food loss, both in developed and developing countries [30]. However, the reality of the places where food is sold can be very different, depending on the country. In Brazil, the sale of both wholesale and retail food products is known as Brazilian Supply Centers [30]. Additionally, there are smaller retail markets that sell to urban populations in smaller quantities [30]. However, these smaller markets, which are the main source of supply of vegetables for the population, present problems in terms of infrastructure and functionality, contributing to substantial food loss [30]. In these locations, foods such as fruit and vegetables, transported in boxes or plastic bags, are left on the floor until they are placed at the point of sale, and only one of the markets has a pre-cleaning unit to remove impurities before displaying them on sales shelves [30]. Additionally, newly arrived fruits and vegetables are often placed on top of existing ones, contributing to their deterioration, either due to overload or lack of replenishment order [30]. Hygienic and sanitary conditions in these places are sometimes unsatisfactory, contributing to the proliferation of microorganisms and consequent loss of food and health risks [130]. Thus, the main causes of loss in these locations, as indicated by sellers, consist of the deterioration of fruits and vegetables due to poor conservation and storage conditions, which are usually carried out at room temperature in the sales area [30]. Excessive handling by the customer in an attempt to assess freshness, associated with the high temperature and sensitivity of this type of food, was another cause cited [30]. Thus, the use of refrigeration equipment during retail contributes to maintaining the freshness and quality of fruits and vegetables, as well as reducing contamination by pathogens [131]. However, the use of this equipment is predominant only in developed countries, whose losses are around 5% during retail in the European Union. On the other hand, economic limitations in developing countries contribute to the lack of acquisition of refrigeration equipment, leading to losses of 30% in Latin American countries [6,132,133]. In fact, a study carried out in Nepal reported losses of 67% of fruits and vegetables due to the lack of refrigerated storage equipment, in contrast to the losses in a supermarket in India (5% to 10%), where this equipment existed [134]. Weekly losses of fruits and vegetables in small markets in Brazil are around 9.5 tons per week, with the highest loss rates being for papayas, bananas, peppers and tomatoes [30]. In Ethiopia, tomatoes were the crop with the highest reported losses, with papaya, banana and pepper losses reported at 30.31%, 19.87% and 22.54%, respectively [135]. In Ghana, 45% of the cabbages received were lost, and in Rwanda, 30% of the bananas also ended up being wasted [123]. In Ethiopia, 16% of plantains are lost during retailing, and in Nigeria, 20% of tomatoes are also lost at this stage [1]. One of the strategies adopted by small market vendors is to gradually reduce the price of fruits and vegetables at the end of the day [30]. In Uganda, 8% of plantain production is partially spoiled and sold at reduced prices [136]. Of the food that was not viable for sale, 35% ended up being donated to economically vulnerable individuals, and 9% was used for consumption by vendors and families [30].

In developed countries, the reality is somewhat different. In general, fruit losses vary seasonally, with the highest losses between July and September and the lowest between January and March [137]. A study conducted in Sweden found that the waste of sixteen vegetables sold by retailers varied between 0.4% and 6.3% [12]. In the United States of America, it was found that more than 2.7 million tons of fruits and vegetables are not sold or harvested because they do not meet aesthetic requirements or because of low market prices [35]. In the retail sector, approximately 2% of fresh fruits and vegetables are wasted [12]. Since 1995, the waste of grains and fruits during retail in this country has been decreasing inversely to the waste caused by the consumer, and this is even more evident in vegetables [20]. In the Nordic countries, the lowest waste rates were found for the sale of onions, cabbages and cucumbers, with values of 0.4%, 0.7% and 0.9%, respectively [12]. In contrast, broccoli, strawberries, cauliflower and celery presented higher rates of waste during retail, being 6.3%, 4.8%, 4.7% and 4.7%, respectively [12]. Contrary to expectations, it was also found that products such as apples, tomatoes and onions generate more waste when sold in packaging. The opposite was found for kiwi [12]. The same study also found that there is more food loss in small stores compared to larger stores, with differences of 6.5% vs. 2.3% for strawberries, 7.3% vs. 1.7% for packaged tomatoes and 7.4% vs. 2.4% for celery [12]. Thus, it was found that in retail stores with an inventory system, waste varies between 0.4% and 6.3%, being higher in more sensitive products such as strawberries, broccoli and cauliflower, as opposed to cabbage, apples and onions, which present less waste [12]. Fruit and vegetable waste thus represents 53% of the total monetary value of food loss, with rates ranging from 1.2% to 14.7% [137].

The reasons for losses of fruit and vegetables during retailing mainly consist of apparent defects, namely, 18% were damp, 29% were wilted, 34% were moldy, 39% were overripe, 52% were bruised and 54% had changed color [137]. In the case of packaged products, with just one damaged unit, everything is often discarded, when alternatively, the packaging can be opened and the units in good condition can be sold separately [137].

In supermarkets in the United States, the percentage of fresh food lost ranged from 2.2% to 62.9%, with only fruit being lost between 4.1% and 43.1% [10]. The reasons attributed to these losses were high susceptibility to damage, lack of public awareness of food knowledge and differences in packaging [10]. However, it is estimated that 61% of food waste could be avoided if food handling and storage were improved [12].

Cereal-based foods, such as bakery products, are also quite perishable and produce high rates of waste. In a study carried out in Austria, where data from several supermarkets were analyzed, it was found that the loss of bread and pastries was in the order of 3.99%, corresponding to 2.83% in monetary value [137]. However, the data do not include the returns of unsold bread to bakers because if it did, the waste rate would be 12.6% [137]. In fact, the percentage of bread and pastries returned is 85% of the total food lost, representing a monetary loss of 25% [137]. These losses vary seasonally, with the highest rates of loss of bread and pastries in December, close to Christmas. Bakery products are usually no longer offered for sale after they reach their expiration date, with the expiration date itself being the main reason for bread waste, even when offered at a reduced price (8%) [137]. It has been found that in urban environments, large quantities of bread are returned [137].

**Figure 10:**
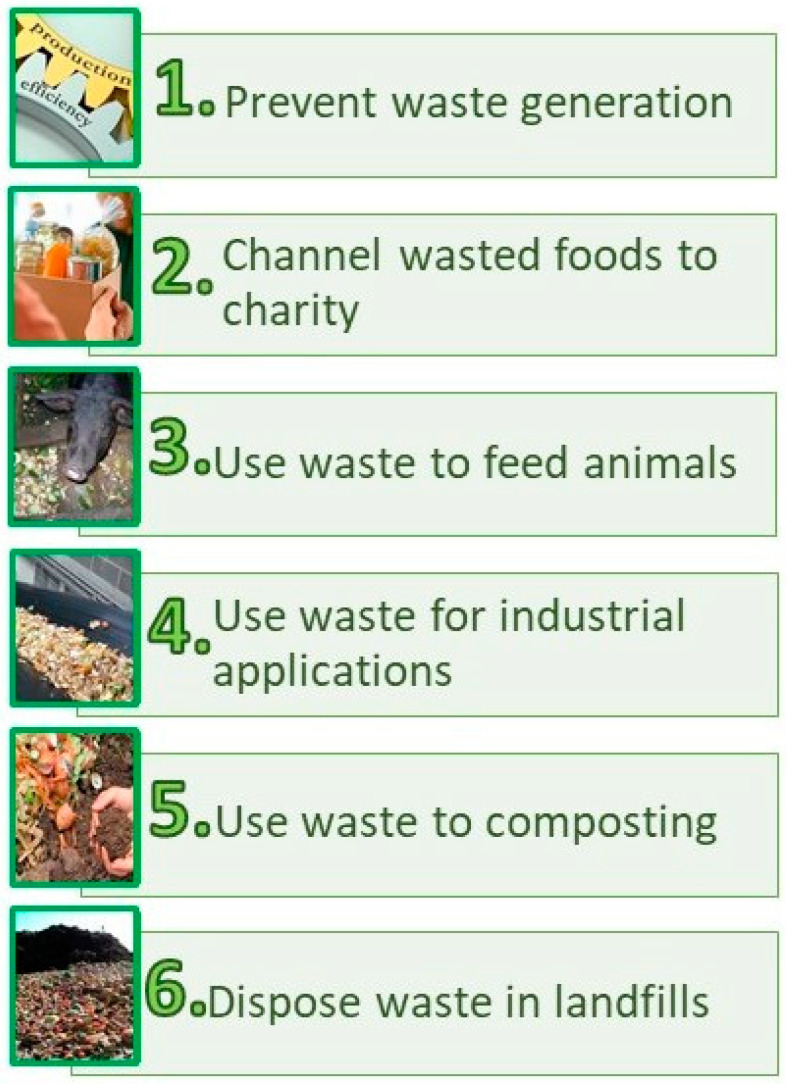
Levels of waste recovery hierarchy.

In 2016, Priefer et al. [138] reviewed the main measures to prevent food losses in Europe, where the importance of donating food to charities was highlighted. Other strategies, such as team sales, reduced-price sales and donations to farms, animal sanctuaries or zoos, were also described [139]. In the United States of America, a pyramid model was developed, called the Waste Recovery Hierarchy, which presents six levels of waste management strategies (Figure 10). The first level aims to prevent waste, the second channels wasted products to charity, the third aims to feed animals, the fourth to apply them in industry, the fifth to use them for composting and the sixth to dispose of them in landfills [140].

Donating food to charity or social services has a social dimension while also minimizing food waste [137]. Lebersorger and Schneider [137] reported that at the supermarket level, only 2.3% of the donated food was fruits and vegetables. On the other hand, a quarter of selling establishments donated 10% of the lost food to social services, but only 1% donated vegetables and fruits, donating 40% of these products [137].

Food donation is an excellent option for products that have been withdrawn from sale but are still safe to consume [32]. In Swedish supermarkets, approximately 840 kg of fruit and vegetables are donated to charitable organizations each month [141]. From an economic point of view, the donation does not allow the retail companies to recover the capital invested, but it does allow them to reduce the generation of waste and associated costs while contributing to a good image of the company itself [137]. Additionally, donation emits less CO_2_ equivalents than would be generated if discarded [137]. In fact, it is estimated that for each kilo of dairy product, 3500 g of CO_2_-equivalents are generated between production, processing, packaging, storage, transportation and marketing [32]. At the same stages, fruits and vegetables generate 320 g of CO_2_-equivalents per kg, and bread and pastries generate 830 g of CO_2_-equivalents per kg [32].

Converting food products that would otherwise be wasted into ones that can later be consumed is also an alternative to value waste. In Sweden, fruit and vegetables that were no longer of a sellable quality but were still suitable for consumption were converted into chutney [141]. Although other ingredients, such as sugar and vinegar, were used in the production process, the generation of waste was avoided [141].

Price reductions for products that would otherwise be discarded, such as approaching expiry dates or minor visual defects, also provide an alternative to food waste generated during retail [141]. In the case of fruits and vegetables with reduced prices, discards were found to be less frequent [137].

## 8. Food Services and Final Consumer

In developed countries, there has been a trend to significantly increase the number of meals from restaurants, regardless of whether they are eaten on-site or as takeaway [10]. Thus, the quantities and types of food waste generated have also evolved [142]. In the catering sector, or in the preparation and cooking of food, the main food waste from foods of plant nature consists of fruits and vegetables, namely grapes, apples, potatoes, tomatoes and citrus fruits [5]. Regarding waste from consumers’ plates, there has been an increase due to exaggerated portions [41]. The proportion and type of leftovers can vary greatly; however, there are foods, such as meat, that are rarely wasted, as opposed to rice and potatoes, that suffer greater waste [143].

Restaurants tend to stock a wide variety of foods so that they can provide customers with a varied menu [29]. In the case of hotels, in addition to food waste, there is also a lack of human resources that leads to difficulties in the way waste is handled, which can hinder recycling [144]. According to the Sustainable Restaurant Association, in the United Kingdom, food waste represents a cost of between 2% and 3% of restaurant turnover [145].

Most food waste occurs at the end of the supply chain in developed countries compared with developing countries [20]. As such, household food waste has a significant impact in terms of environmental problems, as well as economic losses at a global scale [146]. In the United Kingdom, around 70% of food waste after primary production is household waste, and it is estimated that each family throws away food that could have been consumed each week, worth between GBP 4.80 and 7.70. Over the course of a year, this amounts to between GBP 250 and 400, and over a lifetime, it is GBP 15,000–24,000 [145,146]. In Germany, it is also estimated that between 10% and 14% of household food expenditure ends up being wasted [137].

Fruits and vegetables are the most wasted foods in households (about 1/3 of purchased products) [20]. In Germany, 43% of household waste consists of fruit and vegetables [20]. On the other hand, in the United States of America, it was found that between 1995 and 2010, vegetable waste levels decreased significantly [20]. Along with fruit and vegetables, bread and other bakery products and leftovers are also frequently discarded [10]. Household bread waste in the United Kingdom accounted for 32% of all bread purchased [10].

Grains are another food that is wasted in the home, accounting for 7.3% of the 19% of total grain waste in China [147]. In contrast, in poorer countries such as those in sub-Saharan Africa, the percentage of fruit and vegetable waste is lower during consumption (2%) and higher in the previous stages of the food supply chain [1].

Food waste generated by households consists essentially of lignocellulose, polysaccharides and other sugars and can be used as substrate to produce biofuels, pigments, biodiesel, organic acids, oil, functional polysaccharides by microbial fermentation, feed protein, bioplastic, biochar and platform chemicals by thermochemical conversion, cellulose nanocrystals and prebiotic oligosaccharides by chemical and enzymatic hydrolysis [5,102]. Most food waste in households is fed to pets, composted at home, donated to charity or disposed of down the drain [148].

People’s behavior in the store impacts the amount of food wasted, as it is believed that individuals who buy impulsively generate more waste [8]. Frequent shopping and shopping for great amounts of food may be explained by a psychological need to avoid uncertainty and guarantee food security. However, this also contributes to increased food waste [146]. This was observed on a large scale during the COVID-19 pandemic, with consumers buying and storing excessive amounts of food at home [149]. Although household food waste results from purchasing more food than is consumed, it is rarely discarded as purchased [150]. Food passes through several household processes, with the likelihood of disposal increasing at each step [36,151].

The conditions for storage of food at home are an influential factor in household food waste. In 2020, a study carried out by Balan et al. [152] focusing on the behavior of consumers in Germany showed that less than 50% of consumers effectively guarantee the recommended temperatures in their home refrigerators, resulting in 30% to 50% of fresh fruit and vegetables being wasted [152]. Nevertheless, sometimes it is difficult for consumers to understand the appropriate conditions to preserve food, and there is even contradictory information [8]. This is the case with tomatoes, where some entities advise that they be stored in the refrigerator to ensure freshness, but others argue that they should be kept outside the refrigerator to ensure flavor [8,153]. In fact, proper storage of fruits and vegetables can be a challenge for many consumers since some fruits and vegetables end up spoiling if kept at temperatures that are too low (avocado, banana, papaya or melon) [8]. Therefore, fruits and vegetables should be stored between 10 °C and 15 °C in a plastic bag to avoid moisture loss [8]. Additionally, other vegetables (asparagus, carrots, chicory and potatoes) can sprout easily if exposed to sunlight [8]. Any measures addressed to help consumers clarify the correct methods to adopt in order to correctly store the foods at home are beneficial and will certainly contribute to reducing food waste at the household level.

Packaging, whose primary functions are to preserve food and inform the consumer, can also impact food disposal [8]. Characteristics such as disproportionate sizes, difficulties in emptying the packaging and expiration date were factors that dictated food waste [8]. In the United Kingdom, 20% of food is rejected due to the expiration date, being the discard factor in 30% of cases [150]. Although date labels allow us to estimate food safety, they sometimes confuse consumers who believe that they have a similar meaning to expiration [154,155]. Different types of date labels lead to more food being wasted [156]. However, the taste, smell and appearance of food are valid indicators to assess whether or not the food is safe for consumption and contribute to reducing the risk of waste [153].

Preparing food is also an important step to ensure that it does not end up being thrown away. Unintentional cooking without knowing the quantities is a common cause of food waste [153]. Some families prepare more food than is consumed and store the leftovers in the refrigerator or freezer [146]. Many of these foods end up forgotten, uneaten or not visible, resulting in 22.5–46% more waste than in families that regularly consume leftovers [146]. However, other studies indicate that cooking large quantities of food and storing it for later consumption reduces food waste [154,157,158]. Additionally, cooking skills are also important to ensure that food is not discarded due to accidental causes [153]. In fact, families with better cooking skills waste 25% less food than those with lower cooking skills [153].

Consumers do not want to intentionally waste food, as they tend to focus on what is morally and ethically correct, namely food inequality and poverty, but also on the monetary implications [145,159]. Most consumers have an adverse attitude toward waste, but it is individual attitudes that will have an impact on waste [8]. Thus, pessimistic people tend to waste less food [8]. Consumer lifestyles also play a critical role in food waste [146]. Busier, modern lifestyles are associated with a lack of ability to plan meals, prepare quick and convenient meals, order food or dine out, all of which contribute to food waste [149]. One study showed that families that do not dine out have a 21% lower waste rate [146]. Consumers with healthy lifestyles also tend to waste leftovers, as they associate them with being less nutritious and varied, which can contribute to weight gain and have a negative impact on health [150,160].

Household awareness can be raised through information campaigns, with the aim of encouraging the reduction of food waste and informing about the associated consequences [161].

The existence of regulations that motivate consumers to reduce waste, making them aware of the consequences and benefits associated with this behavior, might be advantageous, but people tend to return to their previous behavior as soon as the penalties/benefits are no longer present [162]. Other implementations such as reminders to adopt behaviors that prevent waste, instructions on how to proceed to minimize the problem, providing information on the amount of food wasted/saved and/or comparing it with information from other consumers or changing the conditions of each situation so that people adopt behaviors that prevent food waste have also been successful [150].

## 9. Conclusions

This work highlights, on the one hand, the huge amount of waste produced along the food supply chains while also establishing important measures that are being taken to minimize the negative impacts of this food waste. Food waste is present throughout the entire food life cycle, from the harvesting/collection of raw materials in primary production through processing, distribution and sale up to the moment it reaches consumers’ plates. The impacts of food waste are felt in diverse areas, including environmental impacts, economic effects, influences on society, health, food safety concerns, agricultural activities and waste management.

If, on the one hand, huge amounts of food are discarded on a daily basis, on the other hand, this results in an accumulation of waste in landfills, with risks of contamination of groundwater and largely contributing greenhouse emissions to the atmosphere. More so, these discarded foods could have a role in feeding people who live at social risk.

Some of the discarded products have great potential to be used in the production of new products and in the production of energy, contributing to the circular economy. However, it is crucial to develop and implement effective strategies to reduce food waste. Some countries are focusing on developing environmentally friendly technologies for reprocessing waste. However, consumer awareness of the problem of food waste is an issue that deserves emphasis and will undoubtedly contribute to combating this global problem in the long term. The role of intervening people in the process must be considered, and promoting awareness of the problem through global and local policies can contribute to a more developed conscience of producers, industrials and consumers, helping to decrease the burden of food waste globally.

## 10. Limitations and Future Implications

This work focused on the aspects associated with food waste from different perspectives and along the whole food supply chain. Although it includes a good deal of information and a high number of sources, both scientific and organizational reports, it is possible that other relevant information might have been left out, either because our search did not find it or because it is in the form of documents not readily accessible, including articles whore journals are not subscribed by our institution or reports form organizations that are accessible only if paid for. Although the authors are conscious that some key elements might have been left out involuntarily or due to difficult access, this review contains a very valuable amount of information compiled in a way that includes all the actors along the food supply chain, which is an innovative approach.

The results from this review indicate that promising actions are being taken to minimize food waste and recover valuable products from generated food waste. In this way, new trends and challenges will emerge and arise in the following areas:

- Make agricultural production more effective and produce higher quality products with a lower percentage of materials to discard;
- Make industrial processing more focused on a more rational utilization of raw materials;
- Create more opportunities to recover industrial food waste;
- Implement better distributions and transportation systems, thus minimizing food loss in retail;
- Educate people to reduce food waste at the household and catering levels.

All of these can be achieved based on increased knowledge and the creation of innovative solutions to mitigate the problem of waste generation through proper waste management.

## Figures and Tables

**Table 1:** Composition of food waste streams in recoverable compounds of industrial interest.

Compounds	Waste	References
Phenolics	Wine grape pomace (2–6.5%), Wine grape seeds (4–6%), Citrus peels (5–8%), Apple pomace (0.001–0.29%), Potato peels (0.25–1.2%), Tomato skin (4–15%), Almond hull (2.5–5%)	[69,70,75,76,91,92,93,94,95,96,97,98,99]
Cellulose	Wine grape pomace (27–37%), Wine grape seeds (7%), Citrus peels (9.2–37%), Apple pomace (14–39%), Potato peels (7–40%), Tomato skin (20%), Tomato seeds (16%), Sugarcane bagasse (35–45%), Almond shell (16–41%), Almond hull (9–35%)	[69,70,75,76,91,92,93,94,95,96,97,98,99,100,101,102]
Hemicellulose	Wine grape pomace (26%), Wine grape seeds (24%), Citrus peels (4.2–31.1%), Apple pomace (10–29%), Potato peels (4–14%), Tomato skin (50%), Tomato seeds (11%), Sugarcane bagasse (26–35%), Almond shell (31–36%), Almond hull (7–15%)	[69,70,75,76,91,92,93,94,95,96,97,98,99,100,101,102]
Lignin	Wine grape pomace (16.8–24.2%), Wine grape seeds (49%), Citrus peels (0.54–8.6%), Apple pomace (14–25%), Potato peels (12–32%), Tomato skin (15–25%), Tomato seeds (38%), Sugarcane bagasse (11–25%), Almond shell (29–31%), Almond hull (8–16%)	[69,70,75,76,91,92,93,94,95,96,97,98,99,100,101,102]
Protein	Wine grape seeds (25–40%), Apple pomace (3–7%), Potato peels (10–25%), Tomato seeds (14–40%)	[70,75,76,91,92,93,94,95,103,104]
Limonene	Citrus peels (0.5–4%)	[69,99]
Pectin	Citrus peels (13–42.5%), Apple pomace (8–19%)	[69,70,92,99]
Lycopene	Tomato skin (0.12–0.29%)	[76,93]
Sucrose	Sugarcane molasses (43–45%)	[105]

## Data Availability

No new data were created or analyzed in this study. Data sharing is not applicable to this article.
